# Fungi in the Chilean Altiplano: Analyses of Diversity and Yeasts with Applied Enzymatic Potential

**DOI:** 10.3390/jof11080561

**Published:** 2025-07-29

**Authors:** Jennifer Alcaíno, Claudio Veloso, Maximiliano Coche, Danae Troncoso, Marcelo Baeza

**Affiliations:** Departamento de Ciencias Ecológicas, Facultad de Ciencias, Universidad de Chile, Santiago 7800003, Chile; jalcainog@uchile.cl (J.A.); clveloso@uchile.cl (C.V.); maximiliano.coche@ug.uchile.cl (M.C.); danae.troncoso@ug.uchile.cl (D.T.)

**Keywords:** amplicon metagenome, Altiplano, fungal diversity, culturable yeasts, applied enzymes

## Abstract

Fungal communities in high plateau ecosystems remain understudied despite their crucial roles in soil ecosystems, and yeasts inhabiting extreme regions have potential for industrial and biotechnological applications. We studied the fungal diversity in soils across 14 Chilean Altiplano sites using amplicon-based metagenomics and isolation of yeasts to assess their growth under various conditions and hydrolytic enzyme secretion. Using the metagenomic approach, the Ascomycota and Basidiomycota phyla were found to be the most abundant (85% and 8%, respectively). Unclassified families and genera prevailed at six and ten sites, respectively. At the other sites, the most abundant families included *Cladosporiaceae*, *Teratosphaeriaceae*, and *Sporormiaceae*, and the genera *Oleoguttula*, *Coniochaeta*, and *Peziza*. Biodiversity indices did not correlate with the soil’s geographic origin, organic matter content, humidity, or pH. Most isolated yeasts belong to the *Naganishia*, *Holtermanniella*, and *Vishniacozyma* genera, growing at temperatures ranging from 4 °C to 26 °C. Most isolates could use glucose, sucrose, and maltose as carbon sources and exhibited amylase, esterase, pectinase, and protease activities at 30 °C and below. Our results indicate that the evaluated soil physicochemical parameters do not explain the fungal distribution in the Altiplano and highlight the region as a reservoir of unknown fungi, including yeasts with industrially relevant enzymes.

## 1. Introduction

Fungi play an integral and essential role in sustaining the functionality of soil ecosystems and are among the most extremely tolerant organisms, with highly adaptable lifestyles and ecological and morphological plasticity that enable them to thrive in harsh environments [1,2,3]. However, these microorganisms, especially yeasts, remain underexplored in many regions worldwide, particularly in extreme environments such as cold environments. Yeasts have evolved physiological adaptations to overcome several stressors such as low temperatures, nutrient scarcity, and desiccation, which, in addition to their ecological importance [4,5,6,7], make these characteristics attractive for potential applications in diverse industrial and biotechnological fields [8,9,10,11,12].

The cold environments of the high plateaus, with an average elevation of more than 4000 m above sea level (m.a.s.l.), are the least explored regions of fungal research. The Tibetan Plateau and the Altiplano are the most extensive high plateaus on Earth, and most soil fungal studies have been conducted in the former using next-generation sequencing of amplified taxonomic markers, particularly of the internal transcribed spacer (ITS) region. Metagenomic studies of different regions of the Tibetan Plateau showed that Ascomycota was the most abundant phylum, along with the *Acaulosporaceae*, *Glomeraceae*, and *Nectriaceae* families, and the *Cephalotrichum*, *Discosia*, and *Gymnostellatospora* genera. However, a significant percentage of the sequences (about 30% in some cases) corresponded to unclassified fungal taxa, suggesting the potential discovery of undescribed fungi [13,14,15,16,17,18,19,20,21].

The Altiplano, the second largest plateau in the world, is located in the central Andes of South America and extends from approximately 15° to 21° south latitude. This high-altitude region (average elevation of 3700 m.a.s.l.) is characterized by a climate with low precipitation and pronounced diurnal and interannual thermal variations, including daily and regular minimum soil temperatures, with temperatures below 0 °C (https://agrometeorologia.cl (accessed on 15 June 2024)) [22,23]. The high elevation of this region results in lower atmospheric pressure (about 620 hPa at 4000 m.a.s.l.) and air density compared to sea level, reduced absolute humidity, and low air temperatures, with an intensified diurnal cycle due to surface heating from strong solar radiation and intense radiative cooling at night [22]. Precipitation is concentrated during the austral summer (December–March), when the regional atmospheric circulation favors the advection of moisture from the Amazon basin. Rainfall episodes are primarily associated with convective storms that develop in the afternoon due to intense solar radiation [22]. Microbial studies from the Altiplano are scarce, and most have focused on bacteria [24,25,26,27]. In a study based on the sequencing of fungal ITS regions in the northern Altiplano of Bolivia, Ascomycota was identified as the most abundant phylum, with *Hypocreales* and *Pleosporales* as the most abundant orders, and *Fusarium* and *Didymella* as the most abundant genera [28]. Regarding the Chilean Altiplano, an amplicon sequencing analysis of fungal ITS2 performed in the Salar de Huasco revealed that the majority of sequences were assigned to the phyla Ascomycota, classes *Saccharomycetes* and *Lecanoromycetes*, and Basidiomycota, classes *Pucciniomycetes*, *Microbotryomycetes*, *Cystobasidiomycetes*, and *Tritriachiomycetes* [29].

This study examined the fungal diversity in soils from the Chilean Altiplano using an amplicon metagenomic approach and explored its potential relationship with geographic location and abiotic soil organic matter content, humidity, and pH. Furthermore, yeasts were isolated, identified, and characterized in terms of their growth under different temperatures and carbon sources. The yeast isolates were also tested for hydrolytic enzyme secretion, which may facilitate carbon acquisition from their environment and offer potential for applications in productive fields.

## 2. Materials and Methods

### 2.1. Sampling Locations and Characterization of Soil Samples

**Figure 1:**
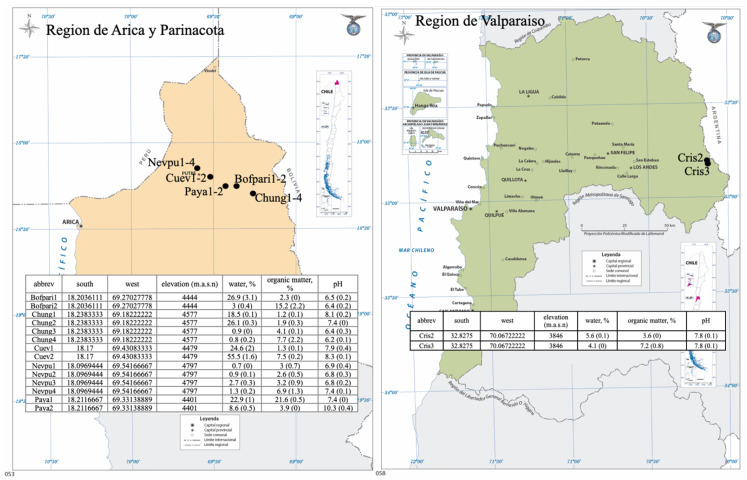
Map of sampled sites. The coordinates and other characteristics of the soil samples are listed in the tables.

In general, bulk soil samples were collected at a depth of 2 to 4 cm adjacent to a plant stem whenever possible, placed in sterile 50 mL tubes, sealed, and stored at 4 °C until arrival at the laboratory, where they were stored at −50 °C until processing. A total of 14 soil samples were collected from different sites on the Chilean Altiplano in the Arica y Parinacota region, all above 4000 m.a.s.l. The samples (Figure 1) included, two samples from the border of Lago Chungará (Chung1 adjacent to Stipa ichu and Chung2 adjacent to *Distichia muscoides*), one sample 30 m away from the border of Lago Chungará (Chung3 adjacent to *Festuca orthophylla*), and one sample, taken 80 m away from border of Lago Chungará (Chung4 adjacent to *Azorella compacta*), two samples from Bofedal de Parinacota (Bofpari1 adjacent to *D. muscoides* and Bofpari2 defecation site of *Lagidium viscacia*), two samples from Mirador Payachatas (Paya1 and Paya2, both adjacent to *Potentilla verna*), two samples from Guardería Las Cuevas (Cuev1 adjacent to *Sagina procumbens* and Cuev2 adjacent to a dead *Sagina procumbens*) and four samples from the slopes of Nevados de Putre volcano (Nevpu1 adjacent to *Nototriche obcuneata*, Nevpu2 adjacent to *A. compacta*, Nevpu3 adjacent to *Parastrephia quadrangularis* and Nevpu4 at 1 m from *P. quadrangularis*). In addition, two soil samples were collected from the Cristo Redentor sector at 3800 m.a.s.l in the Valparaiso region (Crist1 adjacent to an unidentified dead plant and Crist2 adjacent to *Hordeum comosum*). The determination of moisture and organic matter content of the samples was adapted from [30]. Approximately two to three g of soil was incubated at 105 °C for 48 h in a Zhicheng model ZFD-A5040 (Zhicheng, Shanghai, China) oven and then cooled in a drying chamber. The samples were calcined at 550 °C for 2 h in a Nabertherm model L3/P muffle furnace for total organic matter determinations. The mass of the samples was determined using an XP 205 SM-DR (Mettler Toledo, Columbus, OH, USA) analytical balance (±0.00005 g), and the mass difference before and after treatments was used to calculate the moisture percentage and total organic matter. To measure the sample pH, 3 mL of distilled water was added to one g of soil of each sample, vortexed for 1 min, decanted, and the pH was measured using a Hanna Edge pH meter (Hanna Instruments, Woonsocket, RI, USA) (results in the tables of Figure 1).

### 2.2. Extraction of DNA from Soil, Preparation of Libraries, and DNA Sequencing

General molecular biology methods such as DNA quantification, PCR reactions, colony-PCR, agarose gel electrophoresis, and spectroscopic techniques were performed using standardized methods [31].

DNA was extracted from one g of each soil sample using the PowerSoil^®^ DNA Isolation Kit (MO BIO Laboratories Inc., Carlsbad, CA, USA) following the manufacturer’s instructions, except for the disruption step, which was performed by mechanical rupture with 0.5 mm glass beads using a Mini Beadbeater-16 cell disruptor (BioSpec, Bartlesville, OK, USA). The extracted DNA was quantified using a Qubit 4 fluorometer (Invitrogen, Waltham, MA, USA) and sent to Omics2View consulting (Kiel, Germany) for processing, sequencing, and informatics data analysis. The ITS2 libraries were prepared by PCR amplification using fusion primers containing an 8-nt index sequence and the specific primers ITS3 (5′-GCATCGATGAAGAACGCAGC-3′) and ITS4 (5′-TCCTCCGCTTATTGATATGC-3′). The PCR products were purified using Agencourt AMPure XP beads (Beckman Coulter, Brea, CA, USA). The double-stranded PCR products were then heat denatured and circularized by the splint oligo sequence to form single-stranded circular DNA (ssCir DNA). DNA nanoballs (DNBs), each consisting of more than 300 copies, were formed from the ssCir DNA molecules by rolling-cycle replication, loaded into patterned nanoarrays using high-intensity DNA nanochip technology, and sequenced by combinatorial probe–anchor synthesis (cPAS) [32] on an MGI DNBSEQ-G400 in 2 × 300 bp mode (sequencing results in Table S1).

### 2.3. Sequence Processing and Taxonomic Identification

Amplicon sequence variants (ASVs) were determined using the DADA2 pipeline [33] implemented in the R package dada2 v1.28.0. Primer sequences within an edit distance of 3 were detected and removed from the 5′ and 3′ ends of input read pairs using the BBTools package v39.01 [34]. Forward reads with ≤2 expected errors and reverse reads with ≤4 expected errors were retained. The error-corrected reads with a minimum overlap of 20 bp and ≤1 mismatch in the overlap region were assembled into contigs. Chimeric contigs, consisting of two partial sequences of different origins (also known as ‘bimeras’), were removed using the ‘consensus’ procedure implemented in DADA2. The remaining contigs (ASVs) were taxonomically classified using the IDTAXA approach [35] implemented in the R package DECIPHER v2.28.0 [36] using the UNITE database v8.2 [37]. ASVs with classification confidence ≥ 50% were retained. A neighbor-joining phylogenetic tree [38] was constructed from the aligned ASVs using the R package DECIPHER (summary of results in Table S2). This analysis was performed on three subsamples of each soil sample, and only the consistent results across the triplicates were considered.

### 2.4. Data Processing and Statistical Analysis of Data

A normality test was conducted on each data set using Lilliefors, Kolmogorov–Smirnov, Anderson–Darling, and D’Agostino’s K-squared. *p*-values ≤ 0.05 indicate normality. Statistical comparisons were performed using the Kruskal–Wallis test, followed by Dunn’s post hoc test (*p*-value ≤ 0.05). The pandas and SciPy packages [39,40] were run in Python 3.12.3 to perform statistical analyses and to calculate the Shannon index and Bray–Curtis dissimilarity. Co-occurrence networks were constructed using a co-abundance approach based on shared species abundances across sampling sites and a minimum shared abundance method [41,42]. The resulting co-occurrence matrix was used to build an undirected weighted network, which was then analyzed using NetworkX 3.0 [43] to determine the network topology. Plots were generated using the Matplotlib 3.10.0, NumPy 2.2.0, and scikit-learn 1.6.0 packages [44,45].

### 2.5. Isolation and Identification of Yeasts

One gram of soil sample was mixed with one ml of sterile water, vigorously vortexed for 10 min, and allowed to stand for at least 10 min until the coarse particulate material decanted. 200 μL of the aqueous suspension was seeded onto YM (0.3% yeast extract, 0.3% malt extract, 0.5% peptone) agar plates (1.5%) supplemented with 1% glucose (YMG) and 100 μg/mL of each chloramphenicol and ampicillin. Plates were incubated at 4, 15, and 22 °C and visually inspected daily. When a yeast-like colony was spotted, it was immediately transferred to a new plate and incubated at the same temperature as the source plate. Yeast isolates were long-term stored using the gelatin drop method [46,47] and cryopreserved at −80 °C in 30% glycerol. For molecular identification, the ITS1-5.8S-ITS2 region was amplified by colony PCR using the ITS1 and ITS4 universal primers [48] in thermal cyclers (Applied Biosystems). The amplicons were separated by electrophoresis in 1.5% agarose gels, purified using the Wizard^®^ SV Gel and PCR Clean-Up System (Promega, Madison, WI, USA) according to the manufacturer’s instructions, and sent to the DNA sequencing service of the Pontificia Universidad Católica de Chile (https://biologia.uc.cl/investigacion/plataformas/secuenciacion-y-tecnologias-omicas/ accessed on 15 November 2024). The obtained sequences were analyzed and compared by Megablast with the ITS RefSeq Fungi NCBI database and putatively classified at the species level if identity was ≥99.5% with the corresponding hit. Phylogenetic analysis of ITS sequences was performed using the Geneious Prime 2025.1.3 software. Sequences were aligned using Clustal Omega 1.2.3 with default parameters, and the tree was constructed using the Geneious Tree Builder with the following parameters: Genetic Distance Model, Jukes-Cantor; tree build method, neighbor-joining; number of replicates, 1000; and support threshold: 50%.

### 2.6. Evaluation of Isolates for Growth at Different Temperatures and Carbon Sources, and Secretion of Enzymes

Culture turbidity was measured using a WGZ-1A Digital Desktop Turbidimeter (Hinotek, Ningbo, China). To determine the growth curves of each yeast isolate, a colony was suspended in 7 mL of YMG medium in 10 mL volumetric flasks, which were incubated at different temperatures (4, 10, 15, 22, and 26 °C) with vertical rotation at 120 rpm until at least 50 NTU was reached. This culture was then used to inoculate a new 10 mL volumetric flask with 7 mL of YMG medium to reach an initial turbidity of 10 NTU. The obtained growth curves of each yeast isolate cultured at each temperature were compared, and the temperature at which each yeast isolate reached the maximum turbidity was considered as its optimum growth temperature. The range of temperatures at which at least 70% of the maximum turbidity was reached was considered as the suitable temperature range for growth. To analyze growth using different carbon sources, the yeast colonies were inoculated into 10 mL of YMG medium and incubated at 10 or 22 °C until the late exponential phase and then used to inoculate 96-well microplates with 200 μL of YNB medium supplemented with 1% *w*/*v* of various carbon sources (as the sole carbon source), including D-galactose, D-saccharose, L-arabinose, D-cellobiose, D-raffinose, D-maltose, D-trehalose, D-mannitol, D-lactose, D-sorbitol, D-xylose, D-ribose, glycerol, L-rhamnose, D-melibiose, and D-glucose. In each case, the initial A_600_ was adjusted to approximately 0.1, and the plates were incubated at 10 or 22 °C for seven days, with the A_600_ recorded at different time points using a Biotek EPOCH2 microplate reader (Santa Clara, CA, USA).

To evaluate potential extracellular enzyme activities, the yeast isolates were seeded on 1.5% agar plates with YMG or YNB medium supplemented with the appropriate substrate for the tested enzyme activity (Table 1). The plates were incubated at the optimum growth temperature of each yeast isolate until colony development, and each enzyme activity was then evaluated as described in Table 1.

### 2.7. Extraction of Extracellular Proteins and Enzyme Activity Assays

Yeasts were cultured in 100 mL of the appropriate medium (Table 1) until the stationary growth phase and centrifuged at 7000× *g* for 10 min at 4 °C. The supernatant was filtered through a sterile 0.45 μm pore size polyvinylidene fluoride membrane (Millipore, Burlington, MA, USA). Proteins in the supernatant were precipitated by adding ammonium sulfate to reach 80% saturation, incubated at 4 °C for 2 h with agitation, and then centrifuged at 10,000× *g* for 15 min at 4 °C. The resulting pellet was suspended in 0.5 to 2 mL of Tris-HCl buffer (100 mM, pH 7.0) and dialyzed for 12 h at 4 °C against 2 L of the same buffer using a 10 kDa cut-off dialysis bag. Finally, 100 μL aliquots of the protein samples were added to wells cut in the semi-solid media and assayed for enzyme activity, as described in Table 1.

## 3. Results

### 3.1. Fungal Diversity Determined by Amplicon Metagenomics

**Figure 2:**
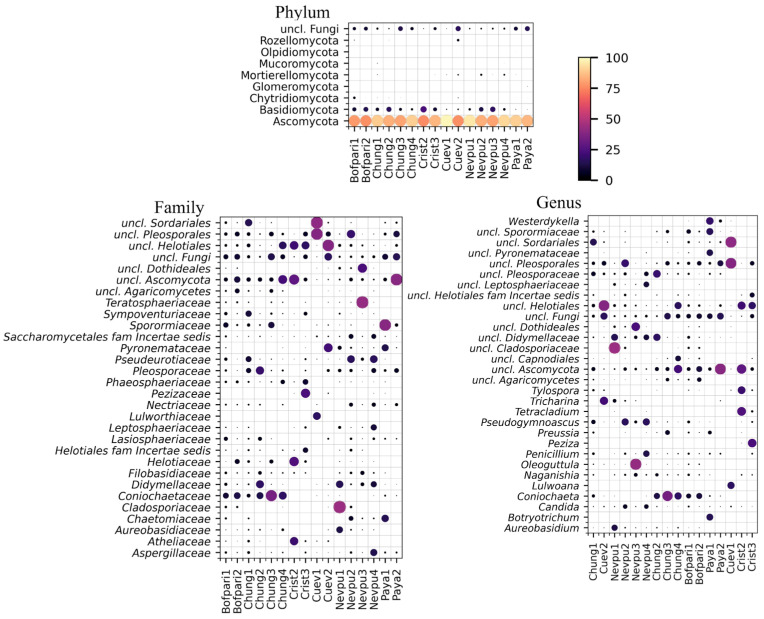
Distribution and abundance of different fungal taxa in the studied sites. The heat map shows the percentages for the 30 most abundant families and genera.

ASVs from the 16 soil samples with successful taxonomical classification (Table S3) are shown in Figure 2 for Phylum, Family, and Genus levels.

Phylum: ASVs were assigned to eight fungal phyla and one unclassified phylum. Ascomycota, Basidiomycota, and unclassified fungi accounted for 99% of all assigned ASVs. Ascomycota was the most abundant at all sites, ranging from 76% at Cristo Redentor Crist2 to 99% at Guardería Las Cuevas Cuev1.

Family: 129 fungal families were identified, along with 43 unclassified ones, with 84% of the total abundance represented by the 30 families. The most abundant families varied across sites, ranging in proportion from 10% to 44%, with *Cladosporiaceae* being the most abundant at Nevados de Putre Nevpu1 (44%), *Teratosphaeriaceae* at Nevados de Putre Nevpu3 (42%), unclassified *Sordariales* at Guardería las Cuevas Cuev1 (40%), unclassified Ascomycota at Mirador Payachatas Paya2 (39%), *Sporormiaceae* at Mirador Payachatas Paya1 (38%), unclassified *Helotiales* at Guardería las Cuevas Cuev2 (38%), and *Coniochaetaceae* at Lago Chungará Chung3 (34%).

Genus: 185 genera were identified, plus 93 unclassified ones, with 77% of the total represented by the 30 genera shown in Figure 2. The genera and sites with the highest percentages were unclassified *Cladosporiaceae* at Nevados de Putre Nevpu1 (44%), *Oleoguttula* at Nevados de Putre Nevpu3 (42%), unclassified *Sordariales* at Guardería Las Cuevas Cuev1 (40%), unclassified Ascomycota at Mirador Payachatas Paya2 (39%) and at Cristo Redentor Crist2 (29%), unclassified *Pleosporales* at Guardería Las Cuevas Cuev1 (38%), unclassified *Helotiales* at Guardería Las Cuevas Cuev2 (38%), *Coniochaeta* at Chung3 (33%), and *Tetracladium* at Cristo Redentor Crist2 (25%).

Globally, the percentage of ASVs assigned to unclassified fungi reached 6% at the phylum level, 41% at the family level, and 57% at the genus level. This reflects the potential for discovering novel fungi from these terrestrial habitats.

**Figure 3:**
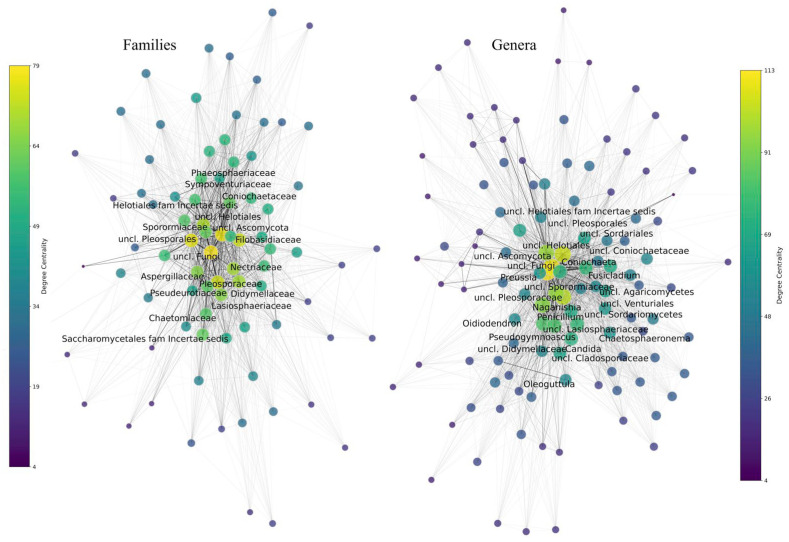
Co-occurrence network of fungal families and genera. The node size and color represent the degree of centrality, while the edge thickness indicates the co-occurrence strength. Only connections above the 25th percentile of co-occurrence values are shown, and only the top 20% of nodes by degree of centrality are labeled.

A co-occurrence network analysis was conducted to identify families and genera that consistently co-occur across sampling locations, focusing on those with a minimum abundance of 1% per site (Figure 3). Based on node degree, the top ten families included *Filobasidiaceae*, *Pleosporaceae*, *Didymellaceae*, *Nectriaceae*, and *Lasiosphaeriaceae*, and five unclassified families. The ten genera with higher node degrees were represented by *Naganishia*, *Penicillium*, and *Pseudogymnoascus*, and by seven unclassified genera.

### 3.2. Fungal Diversity Comparison Among Sites

**Figure 4:**
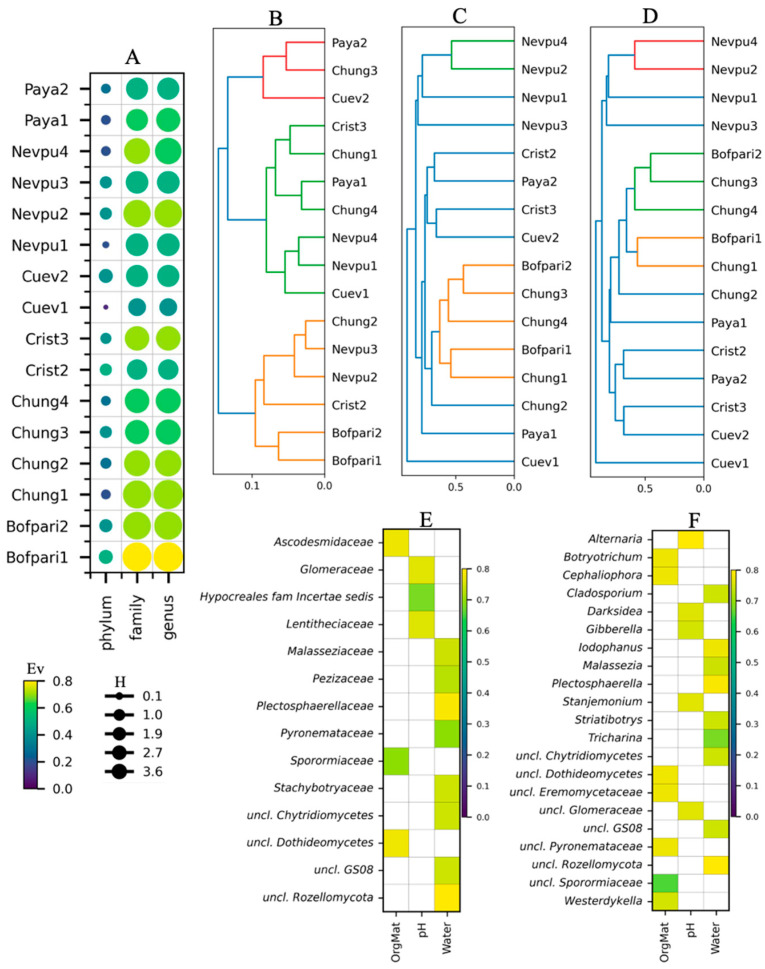
Taxonomic diversity between sites. (**A**) Shannon (H, circle size) and evenness (Ev, circle color) indices at each site for fungal phylum, family, and genus levels. Hierarchical clustering of sites based on Bray–Curtis dissimilarity values for fungal (**B**) phyla, (**C**) families, and (**D**) genera. PCA analysis correlations between soil organic matter (OrgMat), pH, water content (Water), and the abundance of fungal families (**E**) and genera (**F**). Only correlation values of at least 0.7 are shown.

Shannon and evenness indices were calculated for each site, taking into account diversity and abundance data for phyla, families, and genera. A high variation in the Shannon index was observed among sites, with the lowest values in Guardería Las Cuevas Cuev1 (0.1 for phylum, 1.3 for family, and 1.3 for genus) and the highest in Bofedal de Parinacota Bofpari1 (0.8 for phyla, 3.5 for family, and 3.6 for genus) (Figure 4A). A similar trend was observed for the calculated evenness index, with the lowest values also in Guardería Las Cuevas Cuev1 (0.1 for phyla, 0.4 for family, and 0.4 for genus) and the highest in Bofedal de Parinacota Bofpari1 (0.5 for phylum, 0.8 for family, and 0.8 for genus). Bray–Curtis dissimilarity for the same three taxonomic levels was calculated between sites, and hierarchical clustering of the sites was performed based on the results. As shown in Figure 4B–D, the clustering of sites varied depending on the taxonomic level. In some cases, the sites that grouped closely also corresponded to the ones that are geographically closer, such as the Nevados de Putre sites and Lago Chungará with Bofedal de Parinacota sites, which grouped closely at the family and genus levels. However, other sites, such as Cristo Redentor, formed clusters with the more distant sites of Lago Chungará, Guardería Las Cuevas, or Mirador Payachatas. When considering the plant species from which the adjacent soil samples were collected, no correspondence could be found, since *H. comosum* present at Cristo Redentor Crist3 does not correspond to any plant species from the Lago Chungará sites. In addition, soil samples adjacent to *A. compacta* individuals from Lago Chungará Chung4 and Nevados de Putre Nevpu2 clustered separately (Figure 4B–D). In a PCA analysis performed for edaphic parameters and diversity indices of sites, negative correlations (about −0.4) were found between the sample pH and the Shannon and evenness indices for family and genus. The correlations found for water and organic matter content were very low; however, higher correlations were found when the analysis was performed with the abundance data of families and genera found in the sites. Considering correlation values of at least 0.7, the water content of the soil samples correlated with eight families (the highest values were observed with unclassified Rozellomycota) and nine genera (the highest values were observed with unclassified Rozellomycota), pH with three families (the highest with *Glomeraceae* and *Lentitheciaceae*) and five genera (the highest with *Alternaria*), and organic matter with three families (the highest with unclassified *Dothideomycetes*) and seven genera (the highest with unclassified *Dothideomycetes*) (Figure 4E,F).

### 3.3. Isolation and Identification of Yeasts

**Figure 5:**
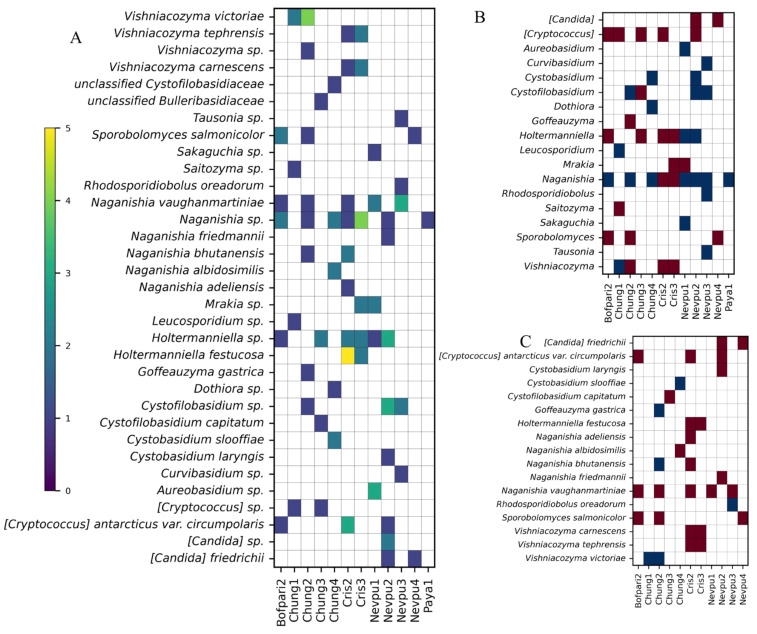
Identification of yeasts isolated from soil samples. (**A**) Heat map representing the number of yeast isolates. Identified cultivable yeast (**B**) genera and (**C**) species, excluding unclassified ones, that matched (blue) and did not match (red) the amplicon metagenome results.

A total of 164 yeast-like colonies were isolated, with the highest number of isolates coming from the samples from Bofedal de Parinacota Bofpari2 (22 isolates), Nevados de Putre Nevpu2 (17 isolates), Lago Chungará Chung4 (15 isolates), Chung1 (14 isolates), Chung3 (14 isolates), Chung2 (13 isolates), and Nevados de Putre Nevpu1 (12 isolates). Molecular identification based on the ITS1-5.8S-ITS2 region sequence allowed the identification of 51 isolates at the species level, 47 at the genus level, and two that could only be classified at the family level (Table S4 and Figure S1). Most of the identified isolates belong to the genera *Naganishia* (27 isolates), *Holtermanniella* (18 isolates), and *Vishniacozyma* (13 isolates). The majority of the identified yeast species were *Naganishia vaughanmartiniae* (eight isolates), *H. festucosa* (seven isolates), *Cystofilobasidium* sp. (six isolates), and *Vishniacozyma victoriae* (six isolates). The most ubiquitous yeast isolates corresponded to those classified as *Naganishia* sp. (isolated from seven sites), *Holtermanniella* sp. (isolated from six sites), and *N. vaughanmartiniae* (isolated from five sites), which were found even in more distant sites such as Lago Chungará and Cristo Redentor (Figure 5A). Isolates corresponding to 19 putative yeast species were found in only one soil sample, and others, such as *H. festucosa*, *Holtermanniella* sp., *Vishniacozyma carnescens*, and *Vishniacozyma tephrensis*, were found only in the Cristo Redentor soil samples. When comparing the culturable yeast genera and species identified (excluding unclassified yeasts) with those from the amplicon metagenomes, noticeable differences were found (Figure 5B,C, and Table S3). Only two of the 23 genera belonging to Ascomycota and nine of the 47 genera belonging to Basidiomycota identified by the metagenomic approach were also cultured. In turn, yeast genera isolated in three soil samples were not detected in the corresponding amplicon metagenomes, and the matches between both approaches ranged from 20% to 50% in 13 samples. Yeast species recovered and identified in seven soil samples were not detected in the corresponding amplicon metagenomes, and in the other 4 samples, the matches at the species level ranged from 60% to 80%.

The isolated yeasts identified at the molecular level were further characterized in terms of their growth temperatures, carbon source assimilation, and production of extracellular enzymes that might have the potential to be applied in industrial processes.

### 3.4. Growth Temperatures of Yeasts

Most of the yeasts exhibited a wide range of temperatures suitable for growth, spanning from 4 °C to 22 °C (11 isolates), 10 °C to 26 °C (nine isolates), and 4 °C to 26 °C (seven isolates). The optimal growth temperature for most of the yeasts was 10 °C (15 isolates, e.g., *Cystofilobasidium capitatum* T3-3, *Naganishia albidosimilis* T4-11, and *V. tephrensis* CR3-12), followed by 22 °C (12 isolates, e.g., *Cystobasidium laryngis* T12-4, *Naganishia bhutanensis* CR2-23, and *V. carnescens* CR3-2), and 26 °C (ten isolates, e.g., *Goffeauzyma gastrica* T2-9, *Naganishia friedmannii* T12-12, and *Rhodosporidiobolus oreadorum* T13-14) (Table S5). Six isolates, including *Cystobasidium slooffiae* T4-2 and *Holtermanniella festucosa* CR2-28, showed an optimum temperature for growth at 4 °C.

### 3.5. Yeast Growth on Different Carbon Sources

**Figure 6:**
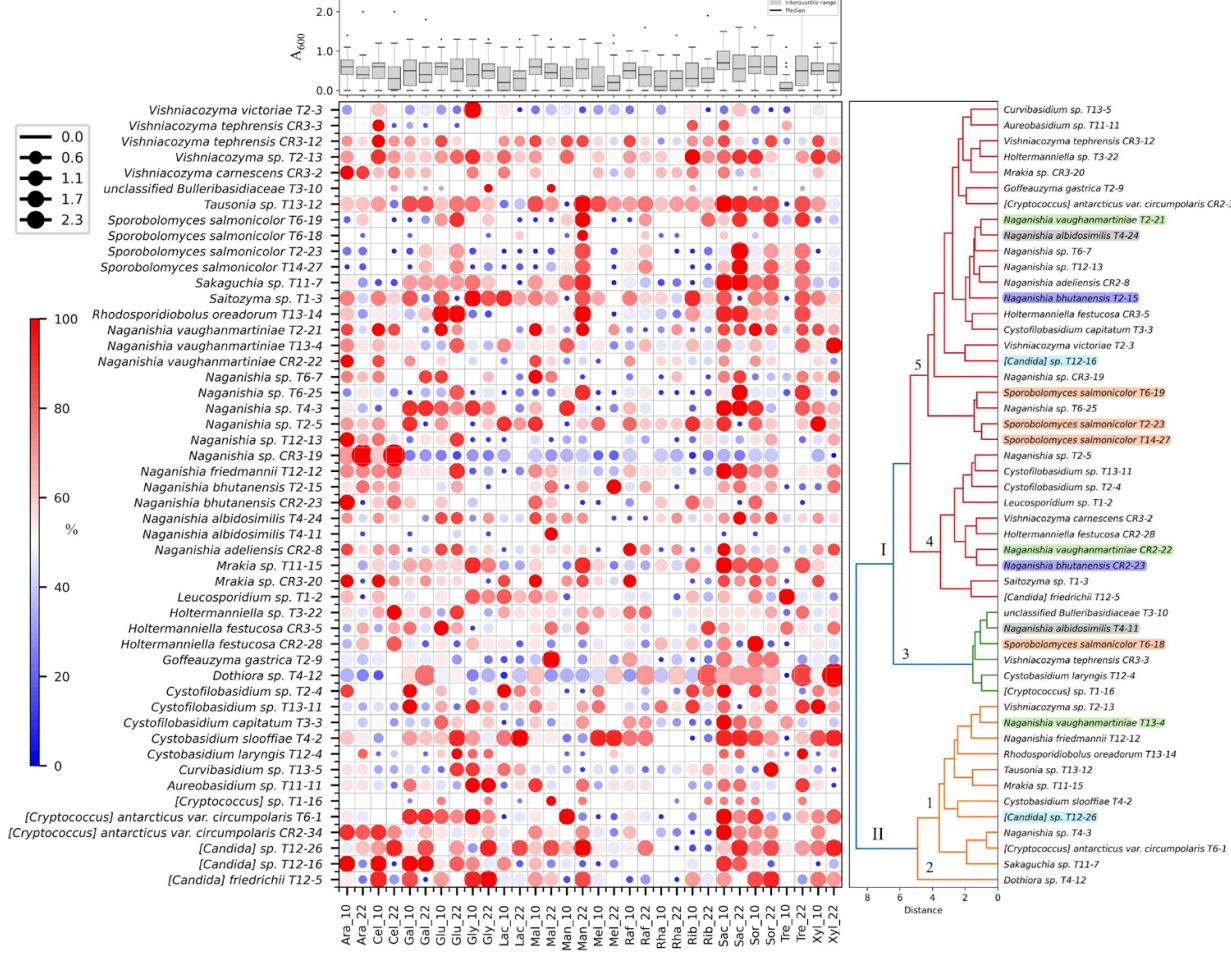
Growth of yeast isolates on different carbon sources. The yeast isolates were cultured at 10 °C (_10) and 22 °C (_22) in minimal medium supplemented with a single compound as the sole carbon source. Growth was measured as the absorbance of the culture at 600 nm. The circle size in the heat map represents the maximum absorbance reached, while the color indicates the percentage relative to the maximum absorbance of each yeast isolate across all analyzed compounds. The box plot shows the distribution of the maximum absorbance of all yeasts in each compound at different temperatures. The dendrogram represents the hierarchical clustering of the yeast isolates based on their maximum growth in the different compounds at both temperatures. Cluster assignments were determined using a distance threshold of 50% of the maximum linkage distance, and yeast isolates with identical ITS sequences are highlighted using the same color. The two main groups (I and II) and the five subgroups (1–5) are indicated. Compound abbreviations: Ara, arabinose; Cel, cellobiose; Gal, galactose; Glu, glucose; Gly, glycerol; Lac, lactose; Mal, maltose; Man, mannitol; Mel, melibiose; Raf, raffinose; Rha, rhamnose; Rib, ribose; Sac, saccharose; Sor, sorbitol; Tre, trehalose; Xyl, xylose.

As 10 °C and 22 °C were the most common optimal growth temperatures, these temperatures were selected to evaluate the ability of the yeasts to grow using different compounds as a sole carbon source (Figure 6). Comparing the growth of all yeasts in each of the 16 tested compounds, the A_600_ medians were generally similar at both temperatures (box plot in Figure 6). Saccharose provided the highest yeast growth (A_600_ median) at both temperatures, while trehalose was higher at 22 °C and maltose and melibiose at 10 °C. Growth on the different carbon sources varied among yeasts, even within the same identified putative species. Considering the top 5 yeasts regarding growth (higher A_600_ median in all compounds), *Dothiora* sp. T4-12, *Tausonia* sp. T13-12, *C. slooffiae* T4-2, and *Saitozyma* sp. T1-3 were consistent at both temperatures, while [*Candida*] *friedrichii* T12-5 and [*Candida*] sp. T12-26 were among the top 5 at 10 °C and 22 °C, respectively. Some yeasts showed marked differences in growth with each compound at 10 °C or 22 °C, such as [*Candida*] *friedrich* T12-5 on galactose, [*Candida*] sp. T12-16 on cellulose, *Dothiora* sp. T4-12 on trehalose, xylose, and ribose, *Mrakia* sp. T11-15, *R. oreadorum* T13-14, and *Sakaguchia* sp. T11-7 on trehalose, *Tausonia* sp. T13-12 on mannitol and trehalose, and *V. victoriae* T2-3 on glycerol.

Since the growth profiles on carbon sources were different among isolates that were putatively identified as the same species, a hierarchical clustering was performed among yeasts with the total growth data (A_600_), resulting in two main groups (Nº I and Nº II) with 12 (Nº I) and 38 (Nº II) members, having two (Nº 1 and Nº 2) and three subgroups (Nº 3, Nº 4, and Nº 5), respectively (dendrogram in Figure 6). No clear clustering pattern was observed, either with respect to the yeast species or the soil sample from which they were isolated. Yeast isolates with identical ITS sequences were also found in different groups. This was the case of *Sporobolomyces salmonicolor* and *N. albidosimilis* isolates, which were present in groups Nº 3 and Nº 5; *N. bhutanensis* in groups Nº 4 and Nº 5; and even some that were separated into the two main groups as [*Candida*] sp. in groups Nº 1 and Nº 5, and *N. vaughanmartiniae* in groups Nº 1, Nº 4, and Nº 5.

### 3.6. Extracellular Enzyme Activity of Yeasts

The ability to secrete hydrolytic enzymes was tested for 19 yeast isolates at their optimum growth temperature. These isolates exhibited between one and seven enzymatic activities, with the highest number of activities detected in *N. vaughanmartiniae* T2-21 and *Naganishia* sp. T6-25 and the lowest in [*Cryptococcus*] sp. T1-16 and *S. salmonicolor* T2-23 (Table 2). The most common enzymatic activities were protease (13 isolates), alkaline phosphatase (12 isolates), lipase (12 isolates), and esterase (10 isolates). No isolates showed laccase or ligninase activities. Selected yeasts displaying higher putative activities at the plate colony assay were cultured in the appropriate liquid medium, and proteins from the cell-free supernatants were concentrated to evaluate the enzyme activity. The enzyme activities observed in plate assays that were confirmed when performing the assay with extracellular protein extracts of the specific isolates included amylase and pectinase in *C. capitatum* T3-3, which showed higher activity at 30 °C; esterase, pectinase, protease, and cellulase in *N. albidosimilis* T4-11, *N. albidosimilis* T4-24, *N. vaughanmartiniae* T2-21, and *V. tephrensis* CR3-12, respectively, with esterase exhibiting higher activity at 10 °C and the others at 30 °C.

## 4. Discussion

There are few studies of fungi inhabiting extreme environments, especially from high-altitude plateaus. Amplicon metagenomic analysis of soils from 14 sites of the Altiplano (northern Chile) and two sites of Cristo Redentor (central Chile) revealed that Ascomycota was by far the most abundant phylum in all the sites studied in this work (76 to 99%), followed by Basidiomycota and unclassified fungi. Six other phyla were detected, with a relative abundance lower than 1%. Similarly, Ascomycota and Basidiomycota were the dominant phyla in soil samples from the Bolivian Altiplano, with abundances ranging from 65% to 84% and 4% to 11%, respectively [28]. The high prevalence of Ascomycota has also been reported in terrestrial habitats of the Tibetan Plateau, reaching more than 80% of the relative abundance in forests, grasslands, and agricultural soils of the Loess Plateau of China [14], and in alpine grasslands of the high-altitude area of the Qinghai–Tibet Plateau [19]. In the Qaidam Basin on the Qinghai–Tibet Plateau, 70% of the sequences were assigned to Ascomycota [16], and Ascomycota and Basidiomycota accounted for more than 82% of the total sequences in Mount Gongga [60]. Although Ascomycota was also identified as the most abundant phylum on the Qinghai–Tibetan Plateau, its relative abundance was lower in this region (48%), followed by Basidiomycota (37%) and Mortierellomycota (12%) [18]. Similarly, in alpine grasslands in the Muli mining area, Ascomycota and Mortierellomycota represented an abundance of approximately 37% and 2%, respectively [21].

Four of our study’s ten most abundant fungal families are unclassified, with *Coniochaetaceae*, *Sporormiaceae*, and *Teratosphaeriaceae* ranking 4th, 6th, and 7th, respectively. In the Qaidam Basin of the Qinghai–Tibet Plateau (QTP), *Nectriaceae* was the dominant family [16], ranked 26th in our study [16]. In terms of fungal genera, among the top 10 most abundant genera found in our study, six corresponded to unclassified fungi, with *Coniochaeta*, *Oleoguttula*, and *Pseudogymnoascus* ranking 5th, 7th, and 8th in abundance, respectively. In a similar study, the genera *Fusarium* and *Didymella* were detected as the most abundant in soils from the Bolivian Altiplano [28], and although these genera were also detected in our study, their abundance was very low, ranking 115th and 136th, respectively. Among the seven most common genera found in Chinese Loess Plateau soils, the most abundant were *Inocybe*, *Penicillium*, and *Humicola* [14], the first of which was not detected in our study, and the others ranked 18th and 42nd in abundance, respectively. Among the 10 most abundant fungal groups detected in our work, four families and six genera corresponded to unclassified fungi, underscoring the limited knowledge about the Altiplano fungal communities, a key component in sustaining these high-altitude ecosystems.

Of the three abiotic soil parameters evaluated in this work, only pH showed some correlation with biodiversity variation, with an inverse correlation of −0.4 with the Shannon and Evenness indices calculated at the family and genus levels. In a region of the Bolivian Andes, the fungal community composition was reported to be mainly influenced by soil pH and phosphorus content [61]. Similarly, soil pH was described as the main factor affecting the fungal diversity of soils along a 2582 m elevation gradient in the eastern Qinghai–Tibetan Plateau [18]. In another study from the Tibetan Plateau region with an elevation gradient from 2813 to 5228 m.a.s.l., the fungal community’s composition was mainly influenced by soil organic carbon and total phosphorus in the low-altitude area and the climatic factors in the high-altitude area [19].

Yeasts play an important ecological role and may have applied potential, especially those inhabiting extreme environments. To the best of our knowledge, no work has reported the isolation of yeasts from high plateaus worldwide. A total of 164 yeast-like isolates were obtained in our work, with a higher number of isolates obtained from soil samples of Bofedal de Parinacota Bofpari2, Nevados de Putre Nevpu2, and the four samples of Lago Chungará. All soil samples were collected near vegetation, such as *A. compacta*, *Stipa ichu*, and *F. orthophylla*, except for Bofedal de Parinacota Bofpari2, which corresponded to a defecation site of the caviomorph rodent *L. viscacia*. Generally, the identified yeasts at the genus or species level have been described in other cold environments, such as those belonging to the genus *Naganishia* and the species *V. tephrensis*. Other yeasts that were putatively identified in this work have a wider distribution, as they have been isolated from soil and plant material, such as *Rhodotorula kratochvilovae*, *C. capitatum,* and *C. laryngis* (sources: Global Catalogue of Microorganisms, https://gcm.wdcm.org/ (accessed on 10 January 2025); MycoBank, https://www.mycobank.org (accessed on 10 January 2025); GlobalFungi Database, https://globalfungi.com (accessed on 10 January 2025)). Among the enzymatic activities tested, amylase, esterase, pectinase, and protease were detected and confirmed to be secreted by yeasts, which have applications in the food, bioremediation, and biofuel industries [62,63,64]. All exhibited activity at 30 °C and below, a beneficial trait for reducing process temperatures to minimize costs and contamination. These results demonstrate the yeasts’ ability to metabolize diverse environmental compounds as carbon sources, underscoring their potential for biotechnological and industrial applications.

In general, there were no apparent differences in the biodiversity of the soil samples according to their geographical origin or according to the vegetation near the sampling point. Even the Bofedal de Parinacota Bofpari2 sample, which mainly corresponds to an *L. viscacia* defecation site, hierarchically clustered together with the other Bofedal de Parinacota site and three sites from Lago Chungará, where samples were taken near different plant species. Furthermore, the two sites from Cristo Redentor are hierarchically grouped separately, each with sites from the Altiplano, although they are the most distant sites, geographically separated from the others by about 1700 km.

Ultimately, it is essential to mention that it is widely recognized that only a limited proportion of microorganisms identified through molecular techniques can be successfully isolated using culturing methods, particularly from extreme environments [65]. This situation was observed in our work; however, it is important to point out that this bias is also observed in the opposite direction. For example, yeast species successfully cultured and identified in our work were not detected in the amplicon metagenomes obtained from the same soil sample from which they were isolated. This was the case of four species of *Naganishia*, two of *Vishniacozyma*, and *S. salmonicolor*. Similar findings of cultured microorganisms not detected by next-generation approaches have been reported in studies of human gut bacterial microbiota [66] and marine rock ponds [67].

## 5. Conclusions

The fungal communities of Altiplano soils studied here are dominated by Ascomycota, particularly by the *Cladosporiaceae* and *Teratosphaeriaceae* families, along with the *Oleoguttula* and *Coniochaeta* genera. However, there were many ASVs associated with unclassified families and genera, highlighting the region’s potential for discovering novel fungal species. The yeast species most frequently isolated belong to the genera *Naganishia*, *Holtermanniella*, and *Vishniacozyma*, and all of the 19 tested yeast isolates showed at least one extracellular enzyme activity, with amylase, esterase, and pectinase being the most common. The amplicon-based metagenomics and culture-based isolation provided complementary views of fungal diversity, as only a subset of sequenced taxa can be cultured, and some culturable yeasts were not detected in metagenomes. Overall, our findings suggest that the Chilean Altiplano is a reservoir of unknown fungi and cold-active enzymes with promising biotechnological and industrial applications.

## Figures and Tables

**Table 1:** Colony assays for the detection of enzyme activity.

Enzyme Activity	Abbrev.	Medium	Reveal Procedure	Positive Results *	Reference
Amylase	Amy	YMG with 0.2% soluble starch	plate flooding with 1 mL of iodine solution	clear halo	[49]
Alkaline phosphatase	AP	YMG with 0.01 M phenolphthalein diphosphate	plate opened and inverted over a container of ammonium hydroxide	pink halo	[49]
Cellulase	Cel	YMG with 0.5% carboxymethylcellulose	plate flooded with 1 mg/mL of Congo Red solution for 15 min, poured off, and then flooded with 1 M NaCl for 15 min	clear halo	[50,51]
Esterase	Est	1% bacto-peptone, 0.5% NaCl, 0.4% CaCl_2_ * 2H_2_O, 1% Tween 80	-	white precipitate halo	[52]
Gelatinase	Gel	YMG with 16% gelatin instead of agar	-	liquefaction of gelatine	[53]
Laccase	Lac	YNB with 1% glucose and 5 mM guaiacol	-	brown halo	[54]
Ligninase	Lig	YNB with 1% glucose and 0.8 g/L lignin		clear halo	[55]
Lipase	Lip	YMG with 1% tributyrin	-	clear halo	[56]
Pectinase	Pec	0.67% YNB medium, pH 7.0 with 1% pectin	flooded with 1% hexadecyltrimethylammonium bromide	clear halo	[49,57]
Phytase	Phy	Medium 0.5% (NH_4_) 2SO_4_, 0.01% NaCl, 0.05% KCl, 0.001% FeSO_4_, 0.01% MgSO_4_.7H_2_O, 0.01% CaCl_2_.2H_2_O, 0.001% MnSO_4_, pH 6.0, 0.5% sodium phytate.	-	clear halo	[58]
Protease	Pro	YMG with 2% casein, pH 6.5	-	white precipitate halo	[50]
Xylanase	Xyl	YMG with 0.5% xylane	-	clear halo	[59]

*, halo observed around the colony.

**Table 2:** Extracellular enzyme activity assays in yeast isolates.

Yeast Isolate	Amy	AP	Cel	Est	Gel	Lac	Lig	Lip	Pec	Phy	Pro	Xyl
*[Candida]* sp. T12-26	-	6.2 (-)	-	-	-	-	-	1.5	-	6	1	+
*[Cryptococcus]* sp. T1-16	-	-	-	-	+++	-	-	-	-	-	7	-
*C. capitatum* T3-3	5 (30)	-	-	-	-	-	-	2.4	4.5 (30)	7	2.2	-
*C. laryngis* T12-4	-	2.5	-	-	+	-	-	-	-	16	2.3	-
*H. festucosa* CR3-5	2	-	3	5 (-)	-	-	-	1.8	-	-	-	-
*Holtermanniella* sp. T3-22	5.5 (-)	-	-	4.7	-	-	-	2.5	-	-	2.6	-
*N. adeliensis* CR2-8	1	4.2	-	4.1	-	-	-	-	-	-	1.6	+
*N. albidosimilis* T4-11	1	3	-	5.4 (10)	-	-	-	2	-	-	-	-
*N. albidosimilis* T4-24	-	3.3	-	5	-	-	-	2.2	4.8 (30)	-	3.6	-
*N. bhutanensis* T2-15	-	-	-	-	-	-	-	3	4.6	-	-	-
*N. vaughanmartiniae* T2-21	1	4	-	3.6	-	-	-	-	3	4	5.4 (30)	+
*Naganishia* sp. T6-25	-	3.8	-	4.9	-	-	-	1.7	-	1	2.2	+
*Naganishia* sp. T6-7	-	4.3	-	4.1	-	-	-	2.2	1.3	4	-	-
*S. salmonicolor* T14-27	-	4	-	-	-	-	-	2.3	-	-	1	-
*S. salmonicolor* T2-23	-	3.2	-	-	-	-	-	-	-	-	-	-
*S. salmonicolor* T6-18	-	4.6 (-)	-	-	-	-	-	-	-	-	1	+
*S. salmonicolor* T6-19	-	2.3	-	-	-	-	-	2.8 (-)	-	6	1	-
uncl. *Bulleribasidiaceae* T3-10	-	-	-	2.4	+++	-	-	-	-	9	9.6	-
*V. tephrensis* CR3-12	2	-	5 (30)	2.4	-	-	-	1.7	-	3	-	-

Amy, amylase; AP, alkaline phosphatase; Cel, cellulase; Est, esterase; Gel, gelatinase; Inv, invertase; Lac, laccase; Lig, ligninase; Lip, lipase; Pec, pectinase; Phy, phytase; Pro, protease; Xyl, xylanase. Values correspond to mm measured from the edge of the colony to the limit of the halo. Assays performed with extracellular protein extracts at several temperatures (10, 15, and 30 °C) and the temperature at which the highest activity was detected is indicated in parentheses. -, no activity detected. In the case of Xyl, the + symbol indicates growth.

## Data Availability

The datasets generated and analyzed during this study are available at the National Center for Biotechnology Information, Bioproject PRJNA1261431, and as Supplementary Materials.

## Supplementary Materials

The following supporting information can be downloaded at https://www.mdpi.com/article/10.3390/jof11080561/s1. Table S1. Sequencing results; Table S2. Tabular summary of the impact of pipeline steps on read pair/ASV counts; Table S3. Taxonomic classification of ASVs. Genera and species that were found as isolates are highlighted in blue; Table S4. Megablast results comparing the ITS1-5.8S-ITS2 sequence from yeast isolates to the ITS RefSeq Fungi database on NCBI; Table S5. Growth temperatures of yeasts; Figure S1. Phylogenetic placement of yeast isolates. The analysis was performed with sequences from the ITS1-5.8S-ITS2 regions using the neighbor-joining method. Bootstrap values greater than 50% are shown (1000 replicates). * Sequences obtained in this work.

## Abbreviations

The following abbreviations are used in this manuscript:

Bofpari1	Bofedal de Parinacota site1
Bofpari2	Bofedal de Parinacota site2
Chung1	Lago Chungará site1
Chung2	Lago Chungará site2
Chung3	Lago Chungará site3
Chung4	Lago Chungará site4
Crist2	Cristo Redentor site2
Crist3	Cristo Redentor site3
Cuev1	Guardería Las Cuevas site1
Cuev2	Guardería Las Cuevas site2
Nevpu1	Nevados de Putre site1
Nevpu2	Nevados de Putre site2
Nevpu3	Nevados de Putre site3
Nevpu4	Nevados de Putre site4
Paya1	Mirador Payachatas site1
Paya2	Mirador Payachatas site2
Ara	arabinose
Cel	cellobiose
Gal	galactose
Glu	glucose
Gly	glycerol
Lac	lactose
Mal	maltose
Man	mannitol
Mel	melibiose
Raf	raffinose
Rha	rhamnose
Rib	ribose
Sac	saccharose
Sor	sorbitol
Tre	trehalose
Xyl	xylose
