# Supplementary figures and images for: Fungi in the Chilean Altiplano: Analyses of Diversity and Yeasts with Applied Enzymatic Potential
Source: J Fungi (Basel). 2025 Jul 29;11(8):561. doi: 10.3390/jof11080561 (PMC12387783; doi:10.3390/jof11080561)

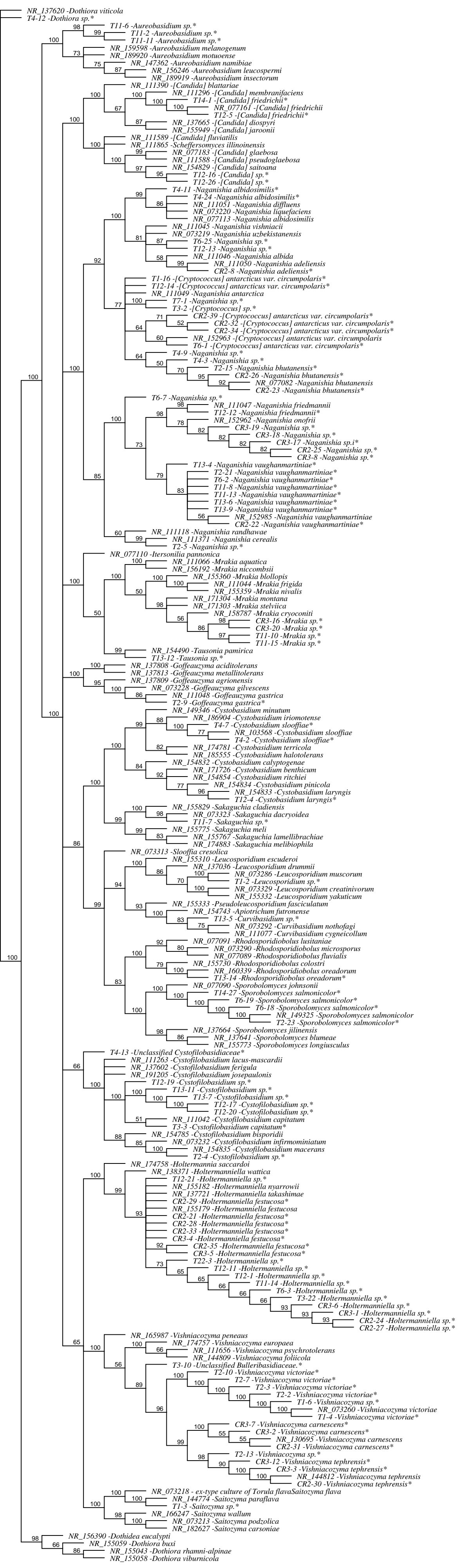

Supplement: Supplementary file 1 [file jof-11-00561-s001.zip › figs1.pdf]
